# Von Jacksch Anemia

**Published:** 1911-12

**Authors:** 


### VON JACKSCH ANEMIA.

Dr. E. P. A. Ficklen, of New Orleans, in the September, 1911 issue of the *N. O. Med. & Surg. Jour.*, reports a case of this rare condition, as follows: The oldest sister of the patient died at the age of nine months with the same symptoms as the patient. Three other children healthy. Patient born of Sicilian parents, at term, seven months old, healthy up to age of two months, then gradually developing pallor and hebetude. No emaciation nor edema nor lymphatic enlargement. Broncho-pneumonia. Liver firm, not tender, extending to umbilicus in median line. Spleen extended 2½ inches below costal arch. Death five days after first examination by reporter, no necropsy.

*Blood Examination:* Hemoglobin, 10% (Tallquist scale).

| | |
|---|---|
| Color index | .2 |
| Red cells per c. m. m | 2,380,000 |
| White cells per c. m. m | 250,000 |

*Differential Count:*

| | |
|---|---|
| Small lymphocytes | 67% |
| Large lymphocytes | 10% |
| Large mono nuclears | 3% |
| Basophiles | 2% |
| Neutrophiles | 16% |

The report also remarks: "Apparent leucocytosis, poikilocytosis, polychromatophilia, anisocytosis, normoblasts, and megoblasts. A few myelocytes, all of them neutrophilic, were seen in one specimen. In several specimens, normoblasts undergoing mitosis, were found."

